# Clinical and genetic analyses of 17 Chinese patients with glycogen storage disease type IXc

**DOI:** 10.1186/s13023-025-04178-1

**Published:** 2025-12-25

**Authors:** Chengkai Sun, Taozi Du, Yu Xia, LuLu Jiang, Manqing Sun, Lili Liang, Kaichuang Zhang, Yi Yang, Yuning Sun, Ruifang Wang, Yu Sun, Bing Xiao, Wenjuan Qiu

**Affiliations:** 1 Department of Pediatric Endocrinology and Genetic Metabolism, Xinhua Hospital, Shanghai Institute for Pediatric Research, School of Medicine, Shanghai Jiao Tong University, Room 803, Sci&Edu Bldg, 1665 Kong Jiang Road, Shanghai, 200092 China; 2 Department of Clinical Genetics, Xinhua Hospital, School of Medicine, Shanghai Jiao Tong University, 1665 Kong Jiang Road, Shanghai, 200092 China

**Keywords:** GSD IXc, PHKG2, Chitotriosidase, Variant

## Abstract

**Background:**

Glycogen storage disease type IXc (GSD IXc) is an ultra-rare disorder impairing liver glycogen degradation, caused by a defect in phosphorylase kinase (PhK) γ subunit in the liver encoded by *PHKG2*. We aim to investigate the clinical, biochemical, genetic, therapeutic, and follow-up characteristics of 17 GSD IXc patients.

**Methods:**

Medical records were retrieved, focusing on clinical (height, complications etc.), biochemical [blood glucose, liver transaminases, chitotriosidase (Chit), etc.], genetic, treatment, and follow-up data for 17 patients (8 males, 9 females) with GSD IXc including 16 pediatric patients and one adult.

**Results:**

Abdominal distension (16/16), hypoglycemia (16/16), muscular weakness (12/16), and short stature (5/16) were among the most common presenting features in 16 pediatric patients. At first visit, all 16 pediatric patients showed increased alanine aminotransferase and aspartate aminotransferase. Elevated gamma-glutamyl transferase, triglyceride, lactate, uric acid and total cholesterol were found in 15/15, 10/14, 7/13, 7/14 and 2/14 pediatric patients, respectively. Creatine kinase levels were within normal range in 14/14 patients. The adult patient was diagnosed with liver cirrhosis on her first visit at 36 years. Five out of sixteen pediatric patients achieved hepatomegaly remission after 8.6 ± 4.0 years of uncooked cornstarch (UCCS). The standard deviation scores for ΔHeight in 16 pediatric patients increased from − 1.76 ± 1.16 to 0.05 ± 1.02 (*p* < 0.0001). Significant improvements were observed in preprandial blood glucose levels and liver transaminases (all *p* < 0.05). Elevated Chit levels at an early stage of therapy decreased with UCCS [44.47 (9.52, 70.03) to 8.22 (6.37, 18.89) nmol/ml/h, *p* = 0.02]. One girl received liver transplantation and her clinical manifestations were greatly improved. Eighteen *PHKG2* variants were identified, including twelve novel variants and one recurrent variant [c.469G > A, p.E157K (allele frequency: 11/34, 32.4%)]. The c.96-11G > A variant was found to cause a 9 bp retention on the right-hand side of intron 1. Patients with biallelic nonnull variants showed better response to UCCS therapy compared to those with null variants.

**Conclusion:**

This study expanded the clinical and variant spectrums of GSD IXc. Chit might be used as a biomarker for monitoring the treatment. Differential response to UCCS therapy based on variant type suggest a genotype-phenotype correlation.

**Supplementary Information:**

The online version contains supplementary material available at 10.1186/s13023-025-04178-1.

## Introduction

Glycogen storage disease type IX (GSD IX) is one of the most common GSDs, with an incidence of 1:100,000 live births [1]. GSD IXc (OMIM 613027) is a severe subtype of GSD IX caused by a deficiency in phosphorylase kinase (PhK, EC 2.7.11.19) and primarily affects the liver. Clinically, GSD IXc manifests as hepatomegaly, hypoglycemia, short stature, elevated liver transaminases, and hypertriglyceridemia. As the disease progresses, a proportion of patients may develop severe liver fibrosis (37.5%) and cirrhosis (6.25%) [1]. To date, approximately 68 cases of GSD IXc have been reported worldwide, including 5 cases previously reported by our center [1–9].

GSD IXc is caused by a defect in PhK γ subunit in the liver, which is encoded by the *PHKG2* gene (OMIM 172471). The human *PHKG2* gene, located on chromosome 16p11.2, consists of 10 exons and spans 9.5 kb. PhK is a heterotetrameric regulatory enzyme composed of the following subunits: α, β, γ, and δ. The γ subunit is encoded by *PHKG2* in the liver. It contains the catalytic site of PhK, which is regulated by the other three subunits and extrinsic calmodulin, potentially contributing to the more severe phenotype observed in GSD IXc. Liver PhK is crucial for glycogen degradation, as it phosphorylates and activates liver glycogen phosphorylase, facilitating the release of glucose-1-phosphate from glycogen. Impaired glycogen breakdown caused by defects in the PhK γ subunit resulting from *PHKG2* gene variation leads to excessive glycogen accumulation in hepatocytes, resulting in associated symptoms.

Liver complications such as fibrosis, cirrhosis, adenoma and hepatocellular carcinoma (HCC) occur more frequently in patients with GSD IXc than in those with other GSD IX subtypes [1], which indicates a worse phenotype. Treatment includes the administration of uncooked cornstarch (UCCS) and a high-protein diet [10]. Currently, there are no reliable biomarkers for predicting the prognosis and assessing the effectiveness of treatments for GSD IXc patients [11]. Chitotriosidase (Chit, EC 3.2.1.14) is an inflammatory marker produced by activated macrophages. Chit activity has been found to be elevated in lysosomal storage disorders, such as Gaucher disease, Fucosidosis, Galactosialidosis, Krabbe disease, Morquio B, Niemann Pick disease, Cholesterol ester storage disease, and GSDs, as well as acquired diseases associated with macrophage activation and inflammation [12–16]. In GSD I, II and IV, elevated Chit levels have been reported, which may be related to the activation of Kupffer cells through glycogen and lipid accumulation [17, 18]. Therefore, Chit activity may be a valuable marker of chronic inflammation in liver GSDs.

GSD IXc is a very rare disease, and the literature consists of mainly case reports or case series with small sample sizes. In the present study, we reported 17 Chinese patients with GSD IXc and delineated their clinical, biochemical, and genetic characteristics, as well as their treatment and follow-up strategies. In addition, we retrospectively analyzed the changes in Chit levels after UCCS treatment.

## Methods

### Patients

Patients who met the following criteria were enrolled: (i) harbored biallelic *PHKG2* variants classified as pathogenic (P) or likely pathogenic (LP), and one variant of unknown significance in one allele and a P or LP variant in another allele; and (ii) had GSD IXc manifestations: hepatomegaly, hypoglycemia and/or short stature. This study received approval from the ethics committee of Xinhua Hospital, Shanghai Jiao Tong University School of Medicine (XHEC-D-2024-025). Written informed consent was obtained from the patients or their legal guardians.

### Study design

A total of 17 eligible participants were included in this study. The collected medical data included (i) demographic information; (ii) age at onset, clinical diagnosis, genetic diagnosis, and last follow-up; (iii) clinical signs/symptoms and laboratory findings; (iv) Chit assay at each follow-up; (v) genetic testing results; (vi) liver biopsy results; and (vii) treatment, including UCCS and liver transplantation. Hepatomegaly was assessed through physical examination and abdominal ultrasound. The stage of liver fibrosis was graded using the METAVIR system in patients who underwent liver biopsy [19, 20]. Liver cirrhosis was assessed using the Child‒Pugh score [21].

The biochemical parameters measured included preprandial blood glucose (PBG), serum alanine aminotransferase (ALT), aspartate transaminase (AST), gamma-glutamyl transferase (GGT), total cholesterol (TC), triglyceride (TG), lactate, uric acid (UA), creatine kinase (CK) and Chit level. The reference ranges for these laboratory parameters are listed in Additional file 1: Table S1. Owing to variations in reference values based on age and sex, values for UA [22] and height (Ht) were converted to standard deviation score (SDS) according to Chinese child growth standards (2009 edition) [23]. The ΔHt SDS was defined as the difference between the SDS of actual height and target height [calculated as (mother’s height + father’s height)/2 ± 6.5 (cm)]. Chit activity was determined according to previously described methods [24]. The Chit assay was administered to the patients following UCCS therapy.

The follow-up period for this study was from February 2014 to August 2024. All patients received UCCS dietary treatment after diagnosis. The treatment included the administration of UCCS (1.5 ~ 2.5 g/kg 4 times daily) and protein (2 g/kg body weight/day). Other treatment included liver transplantation. P9 underwent liver transplantation at the age of 2.9 years after 1.1 years of UCCS therapy and subsequently discontinued dietary therapy.

### Molecular analysis

Genomic DNA was extracted from the peripheral blood of the patients and their parents using a QIAamp DNA Blood Mini Kit (QIAGEN, Valencia, CA, USA). Sanger sequencing was performed in nine patients (P1-6, P8, P15-16) and exome sequencing (ES) was performed in eight patients (P7, P9-14, P17). The genetic test procedures were performed according to previously described methods [25]. The pathogenicity of the variants identified in this study was evaluated according to the guidelines for variant interpretation from American College of Medical Genetics and Genomics (ACMG) [26]. The mRNA *PHKG2* (NM_000294.3) and protein (NP_000016) were used as references.

Chit levels are associated with polymorphic variants in the *CHIT1* (NM_003465.1), and the loss of Chit activity due to homozygosity for a 24 bp duplication in exon 10 of the *CHIT1* is relatively common [14]. Detection of the 24 bp duplication in exon 10 of the *CHIT1* was performed according to the previous reports [27, 28]. Therefore, in the subsequent comparison of Chit levels, we focused on patients who did not have a homozygous 24 bp duplication.

### In silico prediction of splicing and missense variants

The SpliceAI lookup tool [29] was used to evaluate the potential strength of the new/cryptic splicing sites that may result in abnormal splicing for the four variants in the *PHKG2* gene. Missense3D [30] was used to predict the protein structure of the other four missense variants and one common variant, generated by the PyMOL (™) Molecular Graphics System (Version 3.0.5) using the PhK gamma catalytic chain protein crystal structure as a template (PBD code: 2Y7J).

### In vitro mutagenesis and expression

Wild type (WT) *PHKG2* cDNA was constructed using the pcDNA3.1-Flag vector. One recurrent variant (E157K) and other four variants (G173R, F224L, Q249P and R279C) cDNAs were generated from WT pcDNA3.1-PHKG2-Flag via site-directed mutagenesis using the QuikChange Site-Directed Mutagenesis Kit (Stratagene, La Jolla, CA, USA). The variant cDNA was confirmed by sequencing. Plasmids containing variant cDNA or WT control cDNA were transiently transfected into HEK293T cells using PEI. At 48 h post transfection, protein lysates were collected. The expression of the PhK γ subunit protein was analyzed by Western blot (WB). Equal amounts of protein were separated by SDS‒PAGE and transferred to PVDF membranes. The membranes were blocked with 5% BSA and incubated with primary antibodies specific for Flag and β-actin. Appropriate HRP-conjugated secondary antibodies were used for detection, and the bands were visualized using enhanced chemiluminescence. The experiments were replicated three times, and the results are presented with protein banding and densitometry analysis.

### cDNA analysis of the intronic variant of c.96-11G > A

The functional impact of the intronic *PHKG2* variant (c.96-11G > A) was analyzed in vivo through cDNA sequencing. RNA was extracted from the peripheral blood of P14 and a normal control, and cDNA synthesis was performed using the PrimeScript™ RT Reagent Kit (Takara). The cDNA was then used as a template for amplification with two sets of primers: PHKG2-1-F (CAGGATGACGCTGGGACGTGG)/PHKG2-1-R (GCTTCAGATCTCGATGCACA) and PHKG2-2-F (CCGCCAAAGAGTTTTACCAG)/PHKG2-2-R (GGCCACCTTCTCTCTGTGAGAT). The PCR amplification products were subsequently sequenced.

### Statistical analysis

Statistical analysis was conducted using GraphPad Prism 9.5 (GraphPad Software Inc., San Diego, CA, USA; www.graphpad.com). Normally distributed, nonnormally distributed, and categorical variables are presented as the means ± SDs, medians (interquartile ranges), and frequencies, respectively. Age at onset, clinical diagnosis, genetic diagnosis and last follow-up are reported as the medians (range: min ~ max). The paired Student’s t test was used for normally distributed data, and the Wilcoxon test was used for nonnormally distributed data to compare two paired groups. The unpaired Student’s t test was used for normally distributed data, and the Mann‒Whitney U test was used for nonnormally distributed data to compare two independent groups. Chi-square test was used for two categorical variables. The expression of the PhK γ subunit protein by WB was analyzed using one-way ANOVA followed by Dunnett’s multiple comparisons test to compare differences between the mutants and the WT. The level of significance was set at 0.05 (two-tailed).

## Results

### Demographic features

A total of 17 patients (9 females and 8 males) from 17 unrelated families were identified, including 16 pediatric patients and one adult. All 17 patients were born to nonconsanguineous marriage partners. The median ages of onset, clinical diagnosis with GSD, and genetic diagnosis with GSD IXc in 16 pediatric patients were 1.2 (0.6 ~ 2.0) years, 1.5 (0.6 ~ 3.6) years, and 2.3 (1.0 ~ 7.2) years, respectively.

### Initial clinical and biochemical features

Among the 16 pediatric patients, the common presentations at onset included abdominal distension (16/16), hypoglycemia (16/16), muscle weakness (12/16) and short stature (5/16). All pediatric patients presented increased ALT and AST levels; elevated GGT and TG levels were detected in 15/15 and 10/14 patients, whereas mildly elevated lactate, UA and TC levels were detected in 7/13, 7/14 and 2/14 patients, respectively. All 14 patients with recorded CK values were within normal ranges. Two of the pediatric patients underwent liver biopsy (Additional file 1: Table S2).

P17 was diagnosed with cirrhosis by an abdominal computed tomography scan at her initial visit for “dizziness” when she was 36 years old. She subsequently underwent a liver puncture, which revealed cirrhosis (Child‒Pugh A) and glycogen accumulation, and she was subsequently diagnosed with GSD IXc via ES. P17 was queried about her medical history and revealed that she had abdominal distension, muscle weakness, short stature and hypoglycemia as a child, but no laboratory data were documented. Abdominal distension gradually decreased with age, although hypoglycemia and muscle weakness did not significantly improve. She did not receive UCCS therapy until her diagnosis at the age of 36 years. Detailed information on all patients is shown in Table 1 and Additional file 1: Table S3.

**Table 1:** Demographic and clinical information of 16 pediatric patients with GSD IXc

**Demographic information**					
Male/female (n)		8/8			
Age at onset (y)		1.2 (0.6 ~ 2.0)			
Age at genetic diagnosis (y)		2.3 (1.0 ~ 7.2)			
Follow-up duration (y)		8.6 ± 4.0			
**Symptoms**	**pre-treatment**		**post-treatment** ^a^	***p*** **value**	
Hepatomegaly*	16/16		11/16	0.0149	
Muscle weakness*	12/16		6/16	0.0325	
Short stature	5/16		1/16	0.0700	
**Biochemical parameters**	**pre-treatment**		**post-treatment** ^a^	***p*** **value**	
Glucose# (mmol/L) *	2.0 ± 0.6		4.6 ± 0.5	< 0.0001	
ALT (U/L) *	574.2 ± 310.2		137.4 ± 96.0	< 0.0001	
AST (U/L) *	1012.0 ± 634.2		112.0 ± 63.5	< 0.0001	
GGT (U/L, *n* = 15) *	271.0 (122.0, 461.0)		45.5 (26.5, 64.5)	0.0017	
TG (mmol/L, *n* = 14) *	3.2 (2.0, 5.1)		1.4 (1.1, 1.9)	0.0052	
TC (mmol/L, *n* = 14)	4.5 ± 1.9		4.0 ± 1.2	0.1053	
UA SDS (*n* = 14)	2.1 (1.5, 2.9)		0.9 (0.5, 2.7)	0.0963	
Lactate (mmol/L, *n* = 13)	2.9 (1.1, 4.3)		1.5 (1.3, 2.0)	0.0603	
CK (U/L, *n* = 14)	66.5 (46.8, 100.3)		102.0 ( 89.0, 127.0)	0.2871	

Data are shown as n, median (interquartile ranges) (IQR) or mean ± standard deviation (SD). d: day; m: month; y: year. *: clinical symptoms and biochemical parameters with significant difference. #: fasting blood glucose for pre-treatment and pre-prandial blood glucose for post-treatment; ALT: alanine aminotransferase; AST: aspartate transaminase; GGT: gamma-glutamyl transferase; TG: triglycerides; TC: total cholesterol; UA SDS: uric acid standard deviation score; CK: creatine kinase. ^a^: Data of P9 were collected before liver transplantation

### Clinical and biochemical features after dietary treatment

All 16 pediatric patients started UCCS therapy at a median age of 1.5 (0.6 ~ 3.2) years. Eleven out of sixteen pediatric patients (P1-8, 13–14, 16) started UCCS therapy immediately after being diagnosed with GSD, with a median age of 1.4 (0.6 ~ 3.2) years. Four out of sixteen pediatric patients (P9, 11–12, 15) started UCCS after being diagnosed with GSD IXc at a median age of 1.6 (1.3 ~ 2.3) years. One patient (P10) did not begin UCCS until 2.2 years following genetic diagnosis, at the age of 4.8 years. The adult patient started UCCS only following a genetic diagnosis at the age of 36 years. Table 1 and Additional file 1: Table S3 showed the clinical characteristics and biochemical data of all patients following therapy.

The average duration of UCCS therapy (1.5 ~ 2.5 g/kg 4 times daily) was 8.6 ± 4.0 years in 16 pediatric patients. Five out of sixteen patients achieved substantial remission of hepatomegaly after therapy. Six out of the twelve patients with muscle weakness achieved considerable remission. The ΔHt SDS increased from − 1.76 ± 1.16 to 0.05 ± 1.02 (*p* < 0.0001). Two patients reached their final height (ΔHt SDS: P1: -1.02, P2: 0.80). The PBG levels returned to normal (*p* < 0.0001). ALT, AST, GGT and TG levels were significantly decreased (all *p* < 0.05). TC, lactate, UA and CK levels did not significantly change from pretreatment (all *p* > 0.05). P9 underwent liver transplantation at the age of 2.9 years and subsequently discontinued dietary therapy. At the latest follow-up (5.6 years after liver transplantation), the ΔHt SDS of P9 had increased from − 2.25 to 0.15. Her muscle weakness had recovered greatly, and her biochemical parameters had returned to normal range.

P17 had cirrhosis at the time of diagnosis, and P9 underwent liver transplantation after 1.1 years of dietary control; thus, they were excluded from further investigation of Chit levels at each follow-up. Among the remaining 15 patients, five (5/15) patients (P1, P2, P4, P12, P15) carried a homozygous 24 bp duplication in exon 10 of the *CHIT1* (homozygote), nine (9/15) patients were heterozygotes, and one (1/15) was WT. Seven patients [P6 (WT) and P3, P5, P7, P10-11, P13 (heterozygotes)] completed multiple Chit assays during follow-up, and Chit levels [44.47 (9.52, 70.03) nmol/ml/h] were elevated at an earlier stage [2.6 (1.6, 4.8) years after UCCS therapy] but declined to 8.22 (6.37, 18.89) nmol/ml/h at the latest follow-up [8.6 (5.9, 9.6) years after UCCS treatment] (*p* = 0.02). The decrease in the Chit level, along with a significant decrease in the ALT level [(525.0 ± 269.1) vs. (161.1 ± 118.6) U/L] (*p* = 0.0028) and AST level [(1040.0 ± 556.0) vs. (122.1 ± 71.2) U/L] (*p* = 0.0036) after UCCS treatment, may indicate decreased intrahepatic glycogen storage and liver damage.

### *PHKG2* variant spectrum and functional analysis

#### Variant spectrum

**Fig. 1:**
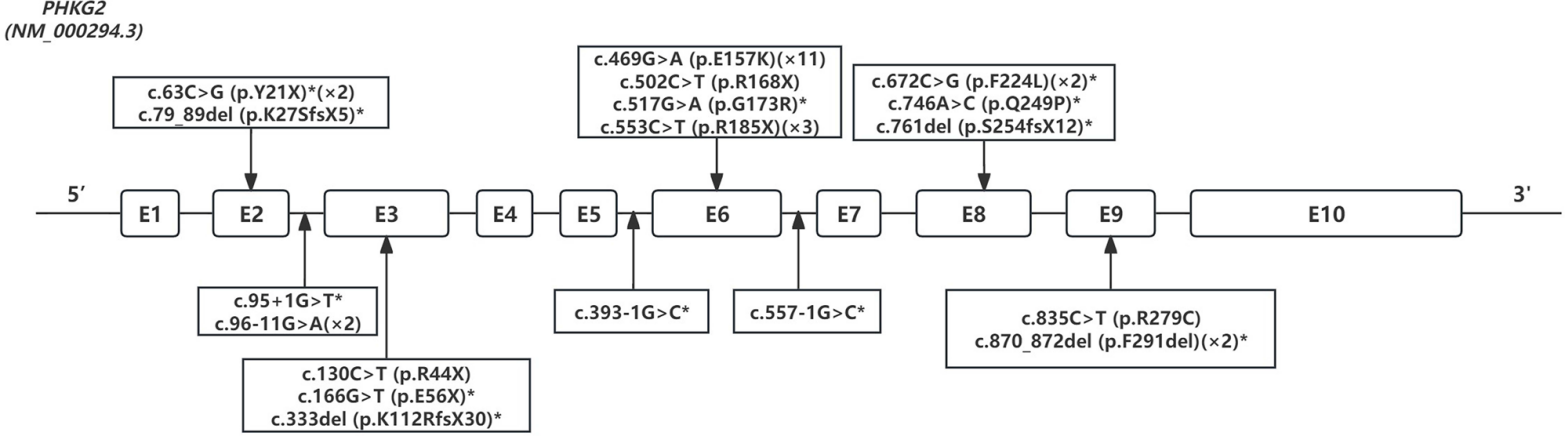
Variants of *PHKG2* gene identified in 17 patients with GSD IXc. The *PHKG2* gene variants were depicted as nucleotide alterations and amino acid alterations per exon/intron. The boxes indicated exons. Novel variants were marked with an asterisk. The suffixes indicated the number of variant alleles

A total of 18 variants in *PHKG2* (NM_000294.3) were identified (Fig. 1), including missense (*n* = 5), nonsense (*n* = 5), frameshift (*n* = 3), in-frame deletion (*n* = 1) and splicing site variants (*n* = 4) in our patients (Additional file 1: Table S4). Twelve of these variants (Y21X, E56X, G173R, F224L, Q249P, F291del, K27SfsX5, K112RfsX30, S254fsX12, c.95 + 1G > T, c.393-1G > C and c.557-1G > C) have not been reported previously. One recurrent variant [c.469G > A, p.E157K (allele frequency: 11/34, 32.4%)] was found in eight patients.

#### In Silico prediction and in vitro expression analysis of the five missense variants

**Fig. 2:**
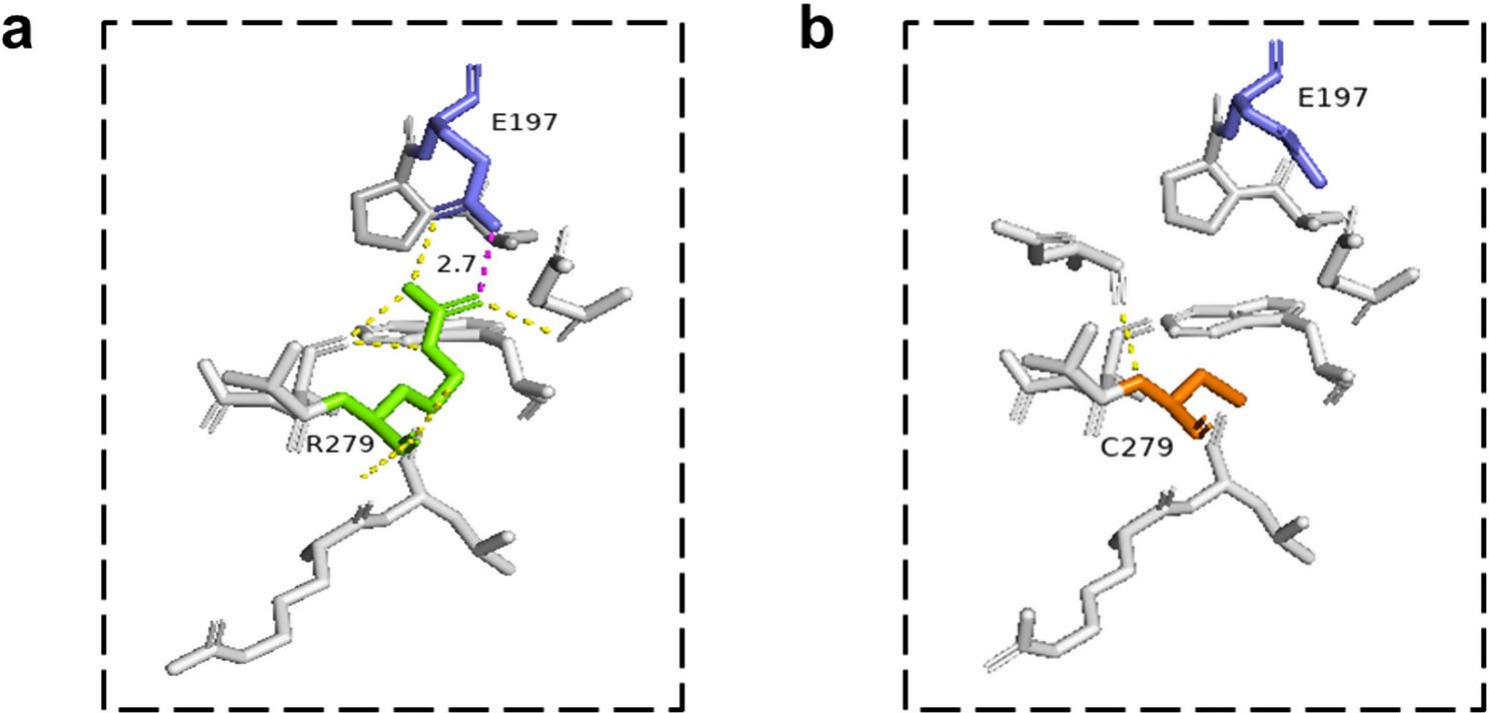
Modelled structure of PhK γ subunit of R279C missense variant. The superimposed structure (PBD code: 2Y7J) of the native arginine (R) (green) and the mutated cysteine (C) (orange) at position 279 of the PhK γ subunit, along with glutamic acid (E) at position 197 (blue). **a** Wild type R279. Salt bridge bond is present with bold purple dashed line between R and E, hydrogen bonds are present with yellow dashed lines in the wild type. **b** R279C variant. The R279C variant disrupts the salt bridge formed by R279 and E197 (distance: 2.7 Å)

In silico prediction for the five missense variants, including one recurrent pathogenic variant (E157K) and other four missense variants (G173R, F224L, Q249P, and R279C), were conducted using Missense3D. The 3D protein models revealed structural instability and changes in the secondary structural features of the R279C variant due to replacement of a buried charged residue [arginine (R), RSA 2.0%] with an uncharged residue cysteine (C) and salt bridge breakage. The other four variants caused no structural damage to the PhK γ subunit (Fig. 2).

**Fig. 3:**
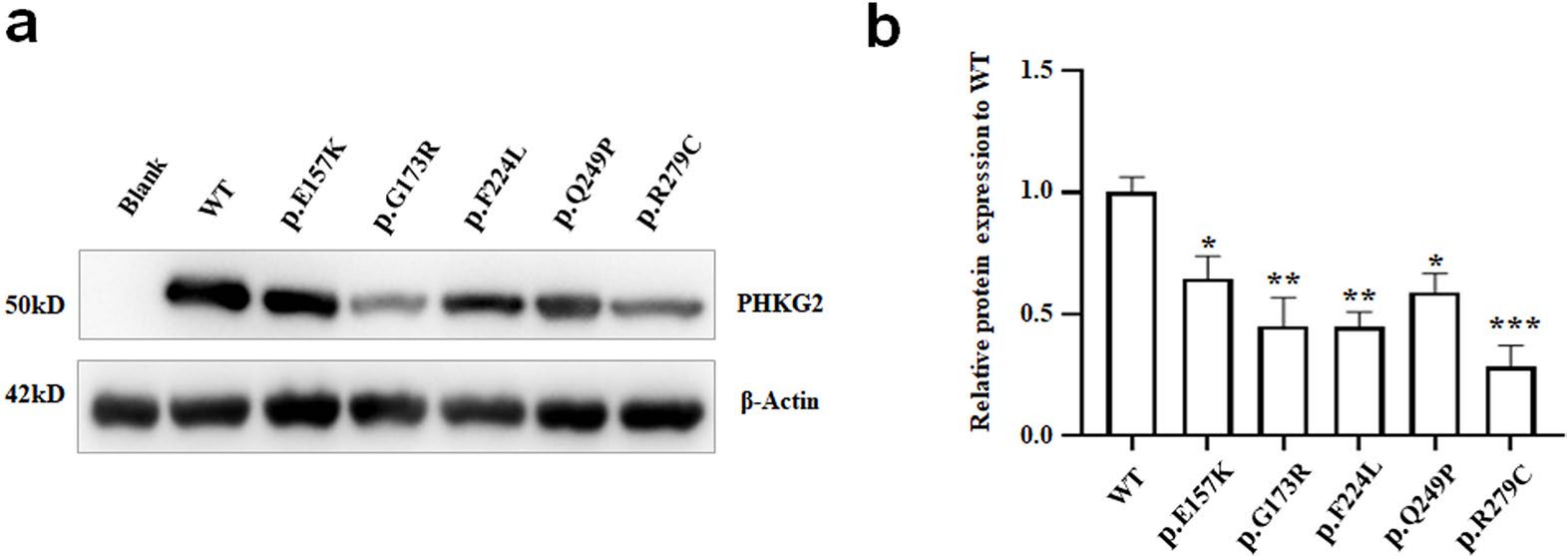
The PhK γ subunit expression of *PHKG2* mutants. **a** Western blot analysis of PHKG2 after transfecting with mutant cDNAs or wild type control cDNA in HEK293T cells; **b** Quantitative analysis of the western blot results in Fig. 3a. * *p* < 0.05, ** *p* < 0.01, *** *p* < 0.001

The PhK γ subunit expression of the five missense variants (E157K, G173R, F224L, Q249P and R279C) was analyzed via WB. Compared with the WT, all five mutants presented significantly reduced expression levels of the PhK γ subunit (all *p* < 0.05). Among the five variants, the expression of the R279C variant was the lowest (Fig. 3).

#### Splicing effect of the intronic variant of c.96-11G > A

**Fig. 4:**
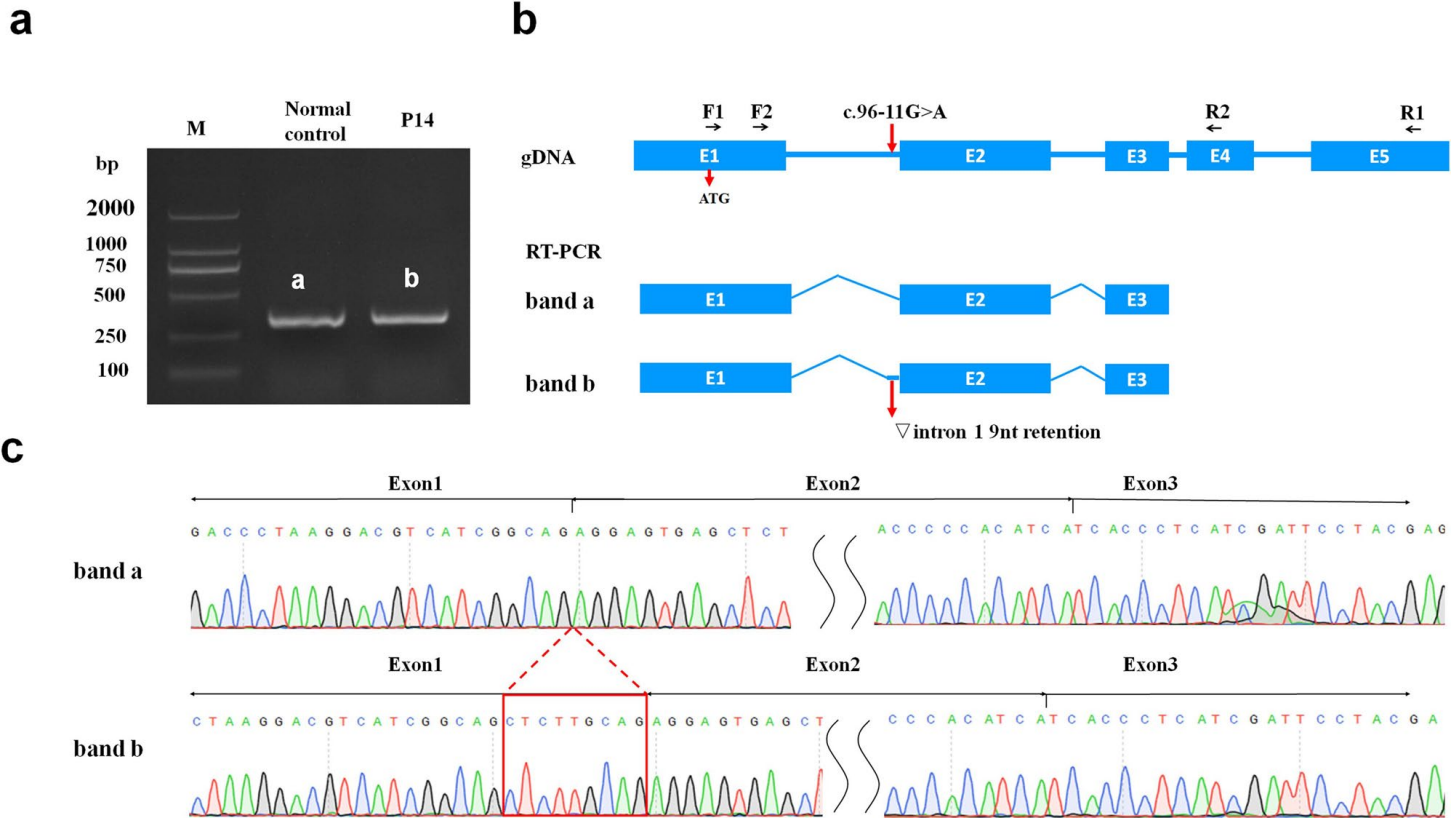
cDNA sequencing analysis of c.96-11G > A**. a** Electrophoresis of RT-PCR products of cDNA (exon1 to 3), labeling the bands obtained by amplification of the normal control (a) and P14 (b); **b** The upper row illustrates the position of the primer. Schematic representation of the transcription of the normal control (band a) and the mutant (band b) *PHKG2* gene. Boxes denote exons, the lines connecting these boxes represent introns. The red arrows indicate the position of c.96-11G > A variant; **c** Sequencing of RT-PCR products confirmed the aberrant retention of a 9 bp fragment from intron 1 adjacent to exon 2

The splicing effects of four splicing variants (c.96-11G > A, c.393-1G > C, c.95 + 1G > T and c.557-1G > C) were assessed through SpliceAI. Three variants (c.393-1G > C, c.95 + 1G > T and c.557-1G > C) were predicted to result in exon skipping, which can be classified as P according to ACMG guidelines. The variant c.96-11G > A was also predicted to produce a new acceptor site, resulting in 9 bp retention. We further analyzed its functional effect via RT‒PCR and cDNA sequencing with RNA extracted from the peripheral blood of P14. The c.96-11G > A variant disrupts normal mRNA splicing of *PHKG2* and leads to a 9 bp retention (c.95_96insCTCTTGCAG, p.Gly31_Arg32insSerSerCys) on the right-hand side of intron 1. This results in an in-frame insertion and a lengthened PhK γ subunit of 409 amino acids (Fig. 4).

#### Genotype‒phenotype analysis

In our study, four patients (P3, P10, P11 and P13) with biallelic null variants (nonsense, frameshift and splicing site variants) were classified as the null variant group. The nonnull variant group included one patient (P14) with biallelic in-frame variants and five patients (P1, P5, P6, P15 and P16) with biallelic missense variants. Patients with nonnull variants were followed up for 8.7 (3.0, 13.7) years, similar to the 7.2 (4.7, 9.6) years reported for those with null variants (*p* > 0.05). ALT/AST levels [96.0 (68.5, 108.8)/79.0 (62.5, 92.3) U/L] in patients with nonnull variants were significantly lower than those in patients with null variants [202.0 (131.3, 336.5)/186.0 (140.8, 215.5) U/L] at the last follow-up (both *p* < 0.05) (Table 2). Patients with nonnull variants were more likely to respond well to UCCS treatment than those with null variants, suggesting a correlation between genotype and phenotype.

**Table 2:** ALT/AST levels at the last follow-up of patients with null/nonnull variants of *PHKG2* gene

Variant type	Patient ID	Allele 1 (paternal)	Allele 2 (maternal)	ALT^*^ (U/L)	AST** (U/L)
Nonnull	P1	c.469G > A (p.E157K)	c.469G > A (p.E157K)	97	67
variant	P5	c.469G > A (p.E157K)	c.835 C > T (p.R279C)	95	72
	P6	c.469G > A (p.E157K)	c.469G > A (p.E157K)	76	86
	P12	c.672 C > G (p.F224L)	c.672 C > G (p.F224L)	98	90
	P14	c.96-11G > A (p.G31_R32insSSC)	c.96-11G > A (p.G31_R32insSSC)	141	99
	P15	c.469G > A (p.E157K)	c.469G > A (p.E157K)	46	49
Null	P3	c.79_89del (p.K27SfsX5)	c.166G > T (p.E56X)	218	211
variant	P10	c.393-1G > C	c.333del (p.K112RfsX30)	186	134
	P11	c.553 C > T (p.R185X)	c.502 C > T (p.R168X)	376	217
	P13	c.557-1G > C	c.130 C > T (p.R44X)	113	161

ALT: alanine aminotransferase, AST: aspartate transaminase. Nonnull variants include in-frame and missense variants; Null variants include nonsense, frameshift and splicing site variants. *: *p* = 0.0190, **: *p* = 0.0095

## Discussion

GSD IXc is an extremely rare subtype of GSD, accounting for 10% (17/170) of GSD IX patients and 1.6% (17/1063) of liver GSD patients in our center. To date, 80 patients with GSD IXc have been reported, including those previously reported and described in this study (Additional file 2), with 25.0% (20/80) originating from China and 23.8% (19/80) from Pakistan. The most prevalent symptoms of liver GSDs are hepatomegaly and hypoglycemia, complicating the differentiation of subtypes based solely on clinical manifestations [31]. In recent years, the expanding use of ES has aided in the early diagnosis of GSDs [3, 31, 32]. UCCS is effective in maintaining blood glucose and improving liver function, but it remains challenging to improve liver complications such as liver fibrosis, cirrhosis, adenoma, and even HCC [1]. The present study involved a retrospective analysis of the diagnosis, genotype, management, and clinical course of 17 Chinese patients with GSD IXc.

Patients with GSD IXc may develop liver fibrosis and cirrhosis, which are more common than those with other GSD IX subtypes [1, 33]. The PhK γ subunit in GSD IXc contains a catalytic site, which is crucial for PhK activity. We retrospectively reviewed 80 patients with GSD IXc, including those previously reported and described in this study (Additional file 2). A total of 27.5% (22/80) of patients had liver fibrosis, 12.5% (10/80) had cirrhosis, 3.8% (3/80) had hepatic adenoma, and 2.5% (2/80) developed HCC. Among the ten patients with cirrhosis, one underwent liver transplantation at 27 years of age due to liver failure. Among the 31 (31/80, 38.8%) patients who underwent liver biopsy, 71.0% (22/31) had liver fibrosis and 32.3% (10/31) had liver cirrhosis. Among the 37 GSD VI patients who underwent liver biopsy, fibrosis was identified in 32.4% (12/37) and cirrhosis in 10.8% (4/37) of the patients [34]. Among the 46 patients with GSD IXa who underwent liver biopsy, 47.8% (22/46) had liver fibrosis, whereas 8.7% (4/46) presented with liver cirrhosis [1]. GSD IXc has a higher incidence of liver fibrosis/cirrhosis. Therefore, PhK deficiency caused by *PHKG2* variants is associated with a more severe phenotype and an increased risk of liver cirrhosis [5, 31, 32]. However, the mechanism of the severe liver phenotype in GSD IXc is still unknown. In our study, P17 did not undergo UCCS therapy until liver cirrhosis was identified at her first visit at the age of 36 years. The remaining 16 pediatric patients in our study who received UCCS therapy at an earlier age of 1.5 (0.6 ~ 3.2) years did not develop liver cirrhosis. Bali DS et al. [35] reported a regression of hepatic adenoma in a GSD IXc patient after UCCS therapy. These results show that early diagnosis and regular therapy could improve patients’ prognoses, and it has previously been documented that active treatment can ameliorate liver cirrhosis in patients with GSD IXa [36]. P9, who underwent liver transplantation showed an obvious remission of symptoms and biochemical parameters, as well as catch-up growth. Therefore, liver transplantation is an effective treatment for GSD IXc [37, 38].

The growth pattern of GSD IXc remains unknown because of its rarity. Our research evaluated the growth status of our patients, with three reaching adult height [ΔHt SDS: 0.05 (-1.02, 0.8)]. Even though the other 14 pediatric patients did not reach their adult height, there was a considerable improvement in height at the latest follow-up. The ΔHt SDS increased from − 1.96 (-2.29, -1.65) at the age of 1.2 (0.6 ~ 2.0) years at the initial visit to 0.10 (-0.38, 0.56) at the age of 9.4 (2.2 ~ 13.3) years at the final follow-up (*p* = 0.0002). These results suggest that the height of GSD IXc patients was not significantly affected. We retrospectively analyzed the height of different subtypes of GSD after UCCS treatment. GSD XI resulted in the most severe growth impairment (mean Ht SDS: -3.5, *n* = 8) [25]. In patients with GSD Ib, the mean ΔHt SDS (− 2.11, *n* = 93) at the last visit did not significantly increase [39]. Patients with GSD Ia showed obvious improvement in height (mean Ht SDS: -0.69, *n* = 25) [40]. While in patients with GSD III/VI/IXa, the mean Ht SDS were − 0.71 (*n* = 13), -0.61 (*n* = 56) and 0.55 (*n* = 51), respectively [41–43]. Therefore, the severity of height impairment in GSDs is as follows: GSD XI, Ib, Ia/III/VI/IX.

Chit is a fully active chitinase expressed by activated macrophages. A marked increase in Chit activity was initially observed in patients with Gaucher disease, and is commonly used in diagnosis, treatment and progression monitoring [44, 45]. Elevated Chit levels have also been observed in Fucosidosis, Galactosialidosis, Niemann Pick B/C, GSD I, GSD II and GSD IV [18, 46]. In chronic liver disease, the accumulation of lipids, glycogen, and fucose in hepatocytes activates liver macrophages (Kupffer cells), resulting in the induction of proinflammatory cytokines and leading to the secretion of Chit [18, 47]. In addition, Kupffer cells activate hepatic stellate cells, which synthesize a variety of extracellular matrix components and induce hepatic fibrosis and ultimately cirrhosis [48, 49]. The Chit level was observed increased in GSD I (21.3 ± 16.4 nmol/h/mL, *n* = 18), GSD II (360 and 420 nmol/h/mL, *n* = 2) and GSD IV (1209 ~ 6088 nmol/h/mL, *n* = 3) [16–18, 50, 51], which may be related to the accumulation of glycogen, resulting in the activation of Kupffer cells [46]. In our study, Chit levels [44.47 (9.52, 70.03) nmol/ml/h] were elevated at an earlier stage (2.6 years after UCCS therapy) but declined to 8.22 (6.37, 18.89) nmol/ml/h at the latest follow-up (8.6 years after UCCS treatment). The decrease in the Chit level, along with a significant decrease in ALT and AST, may indicate decreased intrahepatic glycogen storage and liver damage. Therefore, Chit may be used as a potential biomarker for therapeutic monitoring of GSD IXc.

To date, 48 different variants of the *PHKG2* gene have been reported [2]. In our study, we identified 18 *PHKG2* variants scattered across the entire gene and located mainly in exons 2 to 9, which affected the enzyme activity center in the γ subunit of PhK. One recurrent variant [c.469G > A (p.E157K); allele frequency: 11/34, 32.4%] was identified in eight patients in this study. This variant was common in China (allele frequency: 13/40, 32.5%) and Pakistan (6/38, 15.8%), which is consistent with gnomAD’s findings that p.E157K was more common in East Asian cohorts (allele frequency: 4 × 10^− 5^) and South Asian cohorts (allele frequency: 3 × 10^− 5^) than in the other cohorts (allele frequency: 3 × 10^− 6^), suggesting a potential founder effect in Asia. In general, null variants, including nonsense and splicing variants, can cause the premature appearance of stop codons, resulting in complete loss of gene function via nonsense-mediated mRNA degradation or the production of truncated proteins [5]. While nonnull variants may maintain partial protein function or residual enzyme activity. In this study, the missense variants E157K, F224L and R279C revealed partial PhK γ subunit expression via WB analysis, indicating that these variants may retain residual PhK activity and result in a mild phenotype. Indeed, patients with null variants had considerably higher ALT/AST levels than those with nonnull variants after UCCS treatment, indicating that patients with nonnull variants had a milder phenotype and were more likely to react well to UCCS therapy.

This study has several limitations that should be taken into account. First, most of the GSD IXc patients were preadolescents, and follow-up has not extended into adulthood, perhaps leading to an underrepresentation of long-term complications. Second, since the Chit assay was not accessible in our hospital at the early stage, the majority of patients did not have a baseline Chit level. This study focused only on the results drawn from a comparison of an earlier stage and the final follow-up after UCCS treatment. Finally, this was a retrospective study, thus, some data were missing. Therefore, longer follow-up and larger patient cohorts are necessary to achieve a better understanding of GSD IXc.

## Conclusion

In conclusion, we report on 17 patients with GSD IXc, which helps us better understand their clinical characteristics, variant spectrum, and phenotype-genotype association. Patients with GSD IXc have a more severe liver phenotype. Following regular UCCS therapy, clinical symptoms, biochemical parameters and the prognosis of liver complications could be improved. In addition, the height of patients with GSD IXc was unaffected. Additionally, Chit may be used as a biomarker for monitoring GSD IXc treatment. The response to therapy may vary between patients with biallelic nonnull and null variants, indicating a genotype‒phenotype link in GSD IXc.

## Supplementary Information

Below is the link to the electronic supplementary material.

Supplementary Material 1

Supplementary Material 2

## Data Availability

The datasets used and/or analysed in this current study are available from the corresponding author upon reasonable request.

## Abbreviations

GSD IXc: Glycogen storage disease type IXc
UCCS: Uncooked cornstarch
Chit: Chitotriosidase
PhK: Phosphorylase kinase
HCC: Hepatocellular carcinoma
P: Pathogenic
LP: Likely pathogenic
PBG: Preprandial blood glucose
ALT: Alanine aminotransferase
AST: Aspartate transaminase
GGT: Gamma-glutamyl transferase
TC: Total cholesterol
TG: Triglyceride
UA: Uric acid
CK: Creatine kinase
Ht: Height
SDS: Standard deviation score
ES: Exome sequencing
ACMG: American College of Medical Genetics and Genomics
WT: Wild type
WB: Western blot
