# Supplementary material for: Clinical and genetic analyses of 17 Chinese patients with glycogen storage disease type IXc
Source: Orphanet J Rare Dis. 2025 Dec 25;21:30. doi: 10.1186/s13023-025-04178-1 (PMC12849681; doi:10.1186/s13023-025-04178-1)
Supplement: Supplementary file 1 — Supplementary Material 1 [file 13023_2025_4178_MOESM1_ESM.docx]

**Additional file 1 includes Table S1, Table S2, Table S3 and Table S4.**

**Table S1.** Age-and sex-matched laboratory reference ranges.

| **Laboratory** | **Age** | **Normal range (male)** | **Normal range (female)** |
| --- | --- | --- | --- |
| **Blood glucose (mmol/L)** | All | 3.9~6.1 | 3.9~6.1 |
| **Alanine aminotransferase (U/L)** | 28 days to < 1 year | 8~71 | 8~71 |
|  | 1 to < 2 years | 8~42 | 8~42 |
|  | 2 to < 13 years | 7~30 | 7~30 |
|  | 13 to 18 years | 7~43 | 6~29 |
| **Aspartate transaminase (U/L)** | 28 days to < 1 year | 21~80 | 21~80 |
|  | 1 to < 2 years | 22~59 | 22~59 |
|  | 2 to < 13 years | 14~44 | 14~44 |
|  | 13 to 18 years | 12~37 | 10~31 |
| **Gamma-glutamyl transferase (U/L)** | 28 days to < 6 months | 9~150 | 9~150 |
|  | 6 months to < 1 year | 6~31 | 6~31 |
|  | 1 to < 13 years | 5~19 | 5~19 |
|  | 13 to 18 years | 8~40 | 6~26 |
| **Total cholesterol (mmol/L)** | All | 3.36~6.46 | 3.36~6.46 |
| **Triglyceride (mmol/L)** | All | 0.20~2.31 | 0.20~2.31 |
| **Lactate (mmol/L)** | All | 0.7~2.1 | 0.7~2.1 |
| **Uric acid standardized** | All | -2.0~2.0 | -2.0~2.0 |
| **Creatine kinase (U/L)**  **Chitotriosidase (nmol/ml/h)** | All  All | 39~308  3~18 | 26~192  3~18 |

**Table S2.** Liver biopsy pathologic findings in three cases with GSD IXc.

| **Patients ID** | **Age received liver biopsy** | **Description** | **METAVIR^a^** |
| --- | --- | --- | --- |
| P13 | 6 years | MASSON staining revealed fibrous hyperplasia in the portal regions of the liver, disruption of hepatic lobules, the creation of bridging fibrosis, and pseudolobule development in some locations, with positive PAS staining, PAS (+) | F2 |
| P14 | 2 years 4 months | MASSON staining revealed fibrous cords separating the liver tissue, PAS (+) | F2 |
| P17 | 36 years | MASSON staining revealed hepatic fibroplasia characterized by the development of fibrous septa and pseudolobules in some regions, PAS (+) | F4 |

a: The METAVIR score is a tool used to assess the severity of fibrosis in liver biopsy samples from patients with hepatitis C. It can also be used to initially assess the extent of other liver lesions^[1]^. The score indicates the degree of liver inflammation and the stage indicates the degree of scarring or fibrosis;

**Table S3.** Clinical and biochemical results of 17 patients with GSD IXc at the initial visit and last follow-up.

| **Patient** | **Sex** | **Age at onset/ the last follow-up (y)** | **ΔHt SDS** | **Glucose (mmol/L)** | **ALT (U/L)** | **AST (U/L)** | **GGT (U/L)** | | **TC (mmol/L)** | **TG (mmol/L)** | **UA SDS** | **Lactate (mmol/L)** | **CK (U/L)** | **Hepatomegaly** | **Short stature** | **Muscle weakness** |
| --- | --- | --- | --- | --- | --- | --- | --- | --- | --- | --- | --- | --- | --- | --- | --- | --- |
| **P1** | F | 1.0/16.0 | -1.92/  -1.02 | 0.9/  4.47 | 719/  97 | 1330/67 | | 226/  26 | 0.45/  2.65 | 5.69/4.94 | 3.0/  0.1 | 3.7/  1.0 | 32/  84 | Y/Y | N/N | N/N |
| **P2** | F | 1.5/17.8 | -0.95/  0.80 | 1.30/  5.10 | 429/  25 | 683/  21 | | 159/  24 | 3.26/  1.09 | 2.97/  3.00 | 0.5/  0.5 | 0.1/  1.4 | 46/  NA | Y/N | N/N | N/N |
| **P3** | M | 1.4/11.2 | -3.38/  -0.75 | 2.20/  4.68 | 652/  218 | 1289/211 | | 529/  48 | 8.53/  5.51 | 10.00/  2.21 | 2.3/  NA | 4.6/  3.5 | 64/  NA | Y/Y | Y/N | N/N |
| **P4** | M | 1.1/11.0 | -1.94/  -0.31 | 2.46/  4.03 | 66/  217 | 121/  140 | | 56/  29 | 3.62/  4.64 | 2.17/  1.38 | NA/  0.6 | 3.4/  1.4 | 119/  127 | Y/Y | N/N | Y/Y |
| **P5** | M | 0.6/11.0 | -0.69/  0.94 | 2.00/  4.96 | 740/  95 | 605/  72 | | 40/  53 | 3.80/  3.98 | 3.17/  0.99 | 0.6/  0.5 | 2.1/  1.3 | 73/  122 | Y/Y | N/N | N/N |
| **P6** | F | 1.0/10.1 | 1.74/  2.29 | 2.90/  5.00 | 530/  76 | 1934/86 | | 361/  28 | 6.98/  4.60 | 4.40/  1.48 | 6.9/  3.8 | 2.9/  1.6 | 96/  97 | Y/Y | N/N | Y/N |
| **P7** | M | 1.0/10.7 | -1.75/  0.54 | 3.19/  5.52 | 121/  9 | 241/  20 | | 35/  10 | 4.51/  3.57 | 0.41/  0.70  0.70 | 2.5/  0.2 | 1.2/  2.0 | 60/  106 | Y/N | N/N | Y/N |
| **P8** | F | 1.5/9.5 | -1.99/  0.61 | 1.90/  3.78 | 844/  245 | 1264/155 | | 461/  66 | 4.45/  3.49 | 7.09/  1.16 | 1.8/  NA | 1.0/  NA | 51/  NA | Y/Y | N/N | Y/Y |
| **P9*** | F | 1.6/9.9 | -2.25/  0.15 | 2.30/  3.95 | 1088/189 | 2630/195 | | 271/  38 | 4.52/  3.62 | 2.50/  1.13 | 2.6/  0.9 | 7.4/  1.2 | 45/  246 | Y/N | Y/N | Y/Y |
| **P10** | M | 0.7/8.0 | -1.49/  0.38 | 1.80/  4.30 | 246/186 | 909/  134 | | 665/  60 | 5.74/  3.26 | 3.30/  1.05 | NA/  0.7 | 0.9/  1.4 | 47/  NA | Y/N | N/N | Y/Y |
| **P11** | F | 1.2/7.3 | -1.96/  -0.14 | 1.20/  5.11 | 886/  376 | 1399/217 | | 122/  94 | 4.92/  4.99 | 4.87/  1.83 | 1.9/  4.3 | 1.9/  2.9 | NA/  127 | Y/Y | N/N | Y/Y |
| **P12** | M | 0.7/13.2 | -2.40/  -0.54 | 2.30/  4.20 | 500/  98 | 900/  90 | | NA/  60 | NA/  5.64 | NA/  1.20 | 1.9/  3.1 | NA/  NA | NA/  NA | Y/Y | Y/N | Y/N |
| **P13** | M | 1.0/11.0 | -3.36/  0.29 | 2.20/  3.76 | 371/  113 | 566/  161 | | 336/  179 | NA/  NA | NA/  NA | 1.5/  0.8 | NA/  NA | 69/  89 | Y/Y | Y/N | Y/N |
| **P14** | F | 1.3/8.6 | -2.15/  -2.45 | 2.10/  4.66 | 769/  141 | 991/  99 | | 150/  43 | 4.54/  4.96 | 0.92/  1.86 | 1.5/  1.1 | NA/  NA | 160/  102 | Y/Y | Y/Y | Y/Y |
| **P15** | M | 2.0/5.2 | -1.70/  0.00 | 2.00/  5.20 | 244/  49 | 512/  46 | | 348/  16 | 3.39/  2.90 | 1.60/  1.55 | 2.8/  1.7 | 4.5/  2.0 | 110/  102 | Y/Y | N/N | Y/N |
| **P16** | F | 1.3/12.7 | -1.95/  0.05 | 1.90/  4.60 | 982/  64 | 812/  78 | | 1057/75 | 4.69/  4.57 | 3.42/  0.66 | 4.1/  2.6 | 4.1/  1.8 | 97/  57 | Y/N | N/N | Y/N |
| **P17** | F | 0.0/38.0 | NA/  0.05 | NA/  3.95 | NA/  32 | NA/  33 | | NA/  25 | NA/  NA | NA/  NA | NA/  -0.3 | NA/  NA | NA/  NA | Y/N | Y/N | Y/Y |

*: Data for P9 at the last follow-up is prior to liver transplantation. F: female; M: male. y: years; ΔHt SDS: Δheight standard deviation score, defined as the difference between the SDS of actual height and target height [calculated as (mother’s height + father’s height)/2 ± 6.5 (cm)]; ALT: alanine aminotransferase; AST: aspartate transaminase; GGT: gamma-glutamyl transferase; TC: total cholesterol; TG: triglyceride; UA SDS: uric acid standard deviation score; CK: creatine kinase. NA: not available; N: the patient did not present with the corresponding symptom; Y: the patient presented with the corresponding symptom.

| **Table S4.** *PHKG2* variants in 17 patients with GSD IXc. | | | | | | | | | | |
| --- | --- | --- | --- | --- | --- | --- | --- | --- | --- | --- |
|  |  |  | **Allele 1** | | | | **Allele 2** | | | |
| **Patient ID** | **Paternal origin** | **Maternal origin** | **Nucleotide alteration (NM_000294.3)** | **Amino acid alteration** | **Classification** | **ref.** | **Nucleotide alteration (NM_000294.3)** | **Amino acid alteration** | **Classification** | **ref.** |
| P1 | Hebei | Hebei | c.469G>A | p.E157K | P | [2] | c.469G>A | p.E157K | P | [2] |
| P2 | Hunan | Hubei | c.469G>A | p.E157K | P | [2] | c.553C>T | p.R185X | P | [3] |
| P3 | Sichuan | Sichuan | c.79_89del | p.K27SfsX5 | P | this study | c.166G>T | p.E56X | P | this study |
| P4 | Hubei | Hubei | c.469G>A | p.E157K | P | [2] | c.761del | p.S254fsX12 | P | this study |
| P5 | Guangdong | Guangdong | c.469G>A | p.E157K | P | [2] | c.835C>T | p.R279C | LP | [4] |
| P6 | Jiangsu | Jiangsu | c.469G>A | p.E157K | P | [2] | c.469G>A | p.E157K | P | [2] |
| P7 | Hebei | Hebei | c.553C>T | p.R185X | P | [3] | c.746A>C | p.Q249P | VUS | this study |
| P8 | Jiangsu | Jiangsu | c.870_872del | p.F291del | LP | this study | c.870_872del | p.F291del | LP | this study |
| P9 | Fujian | Fujian | c.63C>G | p.Y21X | P | this study | c.63C>G | p.Y21X | P | this study |
| P10 | Yunnan | Yunnan | c.393-1G>C | / | P | this study | c.333del | p.K112RfsX30 | P | this study |
| P11 | Shandong | Shandong | c.553C>T | p.R185X | P | [3] | c.502C>T | p.R168X | P | [5] |
| P12 | Hubei | Hubei | c.672C>G | p.F224L | LP | this study | c.672C>G | p.F224L | LP | this study |
| P13 | Zhejiang | Zhejiang | c.557-1G>C | / | P | this study | c.130C>T | p.R44X | P | [6] |
| P14 | Hainan | Hainan | c.96-11G>A | p.G31_R32insSSC* | LP | [7] | c.96-11G>A | p.G31_R32insSSC* | LP | [7] |
| P15 | Guizhou | Guizhou | c.469G>A | p.E157K | P | [2] | c.469G>A | p.E157K | P | [2] |
| P16 | Heilongjiang | Heilongjiang | c.469G>A | p.E157K | P | [2] | c.95+1G>T | / | P | this study |
| P17 | Jiangxi | Jiangxi | c.469G>A | p.E157K | P | [2] | c.517G>A | p.G173R | LP | this study |

P: pathogenic; LP: likely pathogenic; /: unknown amino acid alteration. *: Amino acid alteration was verified in this study.

**Reference:**

1. Chowdhury AB, Mehta KJ. Liver biopsy for assessment of chronic liver diseases: a synopsis. Clin Exp Med. 2023;23(2):273–85.

2. Roscher A, Patel J, Hewson S, Nagy L, Feigenbaum A, Kronick J, et al. The natural history of glycogen storage disease types VI and IX: Long-term outcome from the largest metabolic center in Canada. Mol Genet Metab. 2014;113(3):171–6.

3. Waheed N, Saeed A, Ijaz S, Fayyaz Z, Anjum MN, Zahoor Y, et al. Variability of clinical and biochemical phenotype in liver phosphorylase kinase deficiency with variants in the phosphorylase kinase (PHKG2) gene. J Pediatr Endocrinol Metab. 2020;33(9):1117–23.

4. Sperb-Ludwig F, Pinheiro FC, Bettio Soares M, Nalin T, Ribeiro EM, Steiner CE, et al. Glycogen storage diseases: Twenty-seven new variants in a cohort of 125 patients. Mol Genet Genomic Med. 2019;7(11):e877.

5. Davit-Spraul A, Piraud M, Dobbelaere D, Valayannopoulos V, Labrune P, Habes D, et al. Liver glycogen storage diseases due to phosphorylase system deficiencies: diagnosis thanks to non invasive blood enzymatic and molecular studies. Mol Genet Metab. 2011;104(1-2):137–43.

6. Beyzaei Z, Ezgu F, Geramizadeh B, Imanieh MH, Haghighat M, Dehghani SM, et al. Clinical and genetic spectrum of glycogen storage disease in Iranian population using targeted gene sequencing. Sci Rep. 2021;11(1):7040.

7. Bali DS, Goldstein JL, Fredrickson K, Rehder C, Boney A, Austin S, et al. Variability of disease spectrum in children with liver phosphorylase kinase deficiency caused by mutations in the PHKG2 gene. Mol Genet Metab. 2014;111(3):309–13.
